# Supplementary material for: Tumor Radiosensitization by Gene Electrotransfer-Mediated Double Targeting of Tumor Vasculature
Source: Int J Mol Sci. 2023 Feb 1;24(3):2755. doi: 10.3390/ijms24032755 (PMC9917180; doi:10.3390/ijms24032755)
Supplement: Supplementary file 1 [file ijms-24-02755-s001.zip › ijms-2203485-supplementary.pdf]

Supplementary Materials

Table S1. qRT–PCR primers.

	Primer	Primer sequence
Sting	mSTINGF	GTCCTCTATAAGTCCCTAAGCATG
	mSTINGR	AAGATCAACCGCAAGTACCC
Il1β	IL1b-140f	AGTTGACGGACCCCAAAAGA
	IL1b-232r	TGCTGCTGCGAGATTTGAAG
Ifn-β1	IFNb1-241F	TGCCATCCAAGAGATGCTCCAGAA
	IFNb1-364R	AGAAACACTGTCTGCTGGTGGAGT
Tnfα	TNFαF	CCCTCCAGAAAAGACACCATG
	TNFαR	GTCTGGGCCATAGAACTGATG

Table S2. qRT–PCR cycling conditions.

Number of cycles	Temperature	Time
1	50 °C	2 min
	95 °C	2 min
40	95 °C	15 sec
	60 °C	15 sec
	95 °C	15 sec
	60 °C	1 min
	95 °C	15 sec
